# A Parallax Image Mosaic Method for Low Altitude Aerial Photography with Artifact and Distortion Suppression

**DOI:** 10.3390/jimaging9010005

**Published:** 2022-12-25

**Authors:** Jing Xu, Dandan Zhao, Zhengwei Ren, Feiran Fu, Yuxin Sun, Ming Fang

**Affiliations:** 1 School of Computer Science and Technology, Changchun University of Science and Technology, Changchun 130022, China; 2 Zhongshan Institute of Changchun University of Science and Technology, Zhongshan 528403, China; 3 School of Artificial Intelligence, Changchun University of Science and Technology, Changchun 130022, China; 4 Science and Technology on Electro-Optical Information Security Control Laboratory, Tianjin 300308, China

**Keywords:** aerial image, stitching model, feature point extraction, multispectral, image mosaic

## Abstract

In this paper, we propose an aerial images stitching method based on an as-projective-as-possible (APAP) algorithm, aiming at the problem artifacts, distortions, or stitching failure due to fewer feature points for multispectral aerial image with certain parallax. Our method incorporates accelerated nonlinear diffusion algorithm (AKAZE) into APAP algorithm. First, we use the fast and stable AKAZE to extract the feature points of aerial images, and then, based on the registration model of the APAP algorithm, we add line protection constraints, global similarity constraints, and local similarity constraints to protect the image structure information, to produce a panorama. Experimental results on several datasets demonstrate that proposed method is effective when dealing with multispectral aerial images. Our method can suppress artifacts, distortions, and reduce incomplete splicing. Compared with state-of-the-art image stitching methods, including APAP and adaptive as-natural-as-possible image stitching (AANAP), and two of the most popular UAV image stitching tools, Pix4D and OpenDroneMap (ODM), our method achieves them both quantitatively and qualitatively.

## 1. Introduction

At present, when UAVs perform aerial photography in low altitude areas, the field of view is relatively small, thus the captured aerial images cannot contain all the information needed for research. In order to obtain a high-resolution panoramic image, single aerial images are stitched into a wide field of view aerial panorama [1], which provides convenience for the research in meteorological, geological survey, and other fields. Therefore, it is of great significance to carry out the research of UAV aerial image mosaic.

During aerial photography, the UAVs shake due to air flow and other environmental factors, resulting in certain parallax of adjacent aerial images. In this case, if only a single homography matrix is used to align the adjacent images, it is easy to cause the problems of misalignments, artifacts, or due to fewer feature points, leading to mosaic failure of multispectral aerial images. In view of the above problems, we propose an improved APAP image mosaic method, which can stitch seamlessly multispectral aerial images.

## 2. Related Work

The current aerial image mosaic methods are generally divided into two categories, namely, image feature based stitching method and UAV position and attitude information based stitching method [2]. Ruizhe Shao et al. proposed a fast UAV image stitching method that uses the position and attitude information of UAV images to improve the speed of image stitching [3]. This method can quickly find several anchor points to match to stitch images. Compared with the most advanced methods, this method reduces the time cost, but when position and attitude information of aerial images is not available, it cannot be applied. Several authors [4,5,6] proposed UAV aerial images mosaic methods based on SURF, SIFT [7,8,9] or KAZE features. Approach [4] is limited by the time cost of feature points extraction. Methods [5,6] suffer from the problems of ghosting and blurring for parallax image mosaics. In order to improve the efficiency and accuracy of aerial image stitching, Huang et al. [10] proposed to use accelerated nonlinear diffusion (AKAZE) algorithm to match the features of aerial images, and then use the image registration model of As-Projective-As-Possible Image Stitching (APAP) [11] to align aerial images, and finally use Laplace Pyramid Fusion Algorithm [12] to blend overlapping regions. If the error is not properly controlled, the method [10] will cause overall distortions of the mosaic image when the number of UAV aerial images to be stitched is large. While considering improving image alignment, more and more scholars focus on reducing the projection distortion of nonoverlapping regions. Li [13] and Xiang [14] proposed to protect non-overlapping regions to ensure the final mosaic panoramic image more natural.

When most algorithms eliminate the projection distortion of non-overlapping regions, meanwhile, they also destroy the structure information in the images, resulting in the discontinuity of the transition from the overlapping to the non-overlapping regions.

The major work of this paper is as follows

- Align the aerial images with AKAZE algorithm, which takes into account the registration efficiency and preserves the boundary of the region in the image while suppressing noise.
- Introduce the local and the global similarity constraints and focus on the transition from overlapping regions to non-overlapping regions during the registration process.
- Introduce the line protection constraint to deal with the projection distortion of nonoverlapping areas, moreover the geometric structure of the image is also concerned.

## 3. Enhanced Image Mosaic Method Based on APAP

**Figure 1:**
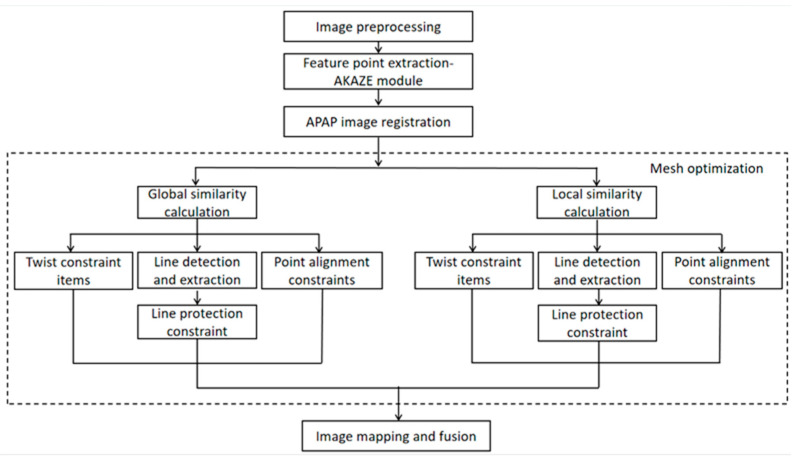
Flow chart of aerial image stitching.

APAP algorithm can solve the problem of artifacts caused by parallax of aerial images, so we propose an aerial image registration approach with improved APAP mesh warp. The algorithm flow is shown in Figure 1. First, preprocess the aerial image, and then extract the feature points of the aerial image through the fast and stable AKAZE algorithm instead of the SIFT algorithm. Then, incorporate the APAP algorithm and improve it. In the registration process, add local and global similarity constraints to reduce the global projection distortion as much as possible. Furthermore, the straight line of the images is detected, and the line protection constraint is added to ensure the structural information of the images to increase the naturalness of panorama. The over overlapped images are not used to create the results. Finally, generate mosaic panoramic image.

1. AKAZE module

KAZE feature is one of most popular multi-scale two-dimensional feature detection and description algorithm in nonlinear scale space [15]. The traditional method is to detect and describe features at different scales by constructing or approximating the Gaussian scale space of the image. However, Gaussian blur does not retain the natural boundary of the object, and smoothing processing is carried out in detail and noise, reducing the positioning accuracy and uniqueness. The larger the Gaussian blur, the greater the loss of local area detection features in the rough scale space. KAZE uses nonlinear diffusion filtering [16,17] to detect and describe two-dimensional features in the nonlinear space, so that the fuzzy part can adapt to the image data, reduce noise, and moreover retain the boundary of the object and obtain uniqueness and positioning accuracy.

AKAZE is an accelerated version of KAZE feature, which uses nonlinear diffusion filtering to build the scale space, introduces an efficient improved local difference binary descriptor (M-LDB), and the descriptor obtained through the AKAZE feature algorithm has rotation invariance, scale invariance, illumination invariance [18,19,20]. The AKAZE detector is a determinant based on the Hessian matrix. The Scharr filter is used to improve the rotation invariance quality. The maximum value of the detector response in the spatial position is picked up as a feature point, which makes the fuzzy local adaptive feature points in the image. The boundary of the area in the subject image is retained while reducing noise. Compared with SIFT and SURF algorithms, AKAZE algorithm is faster.

2. Global similarity constraint

In order to reduce the distortions in the process of image registration, global similarity constraint is added. If there is no global similarity constraint, the final stitching results may be skewed and deformed. We adopt the method in [21] to compute the global similarity constraint. Assume that the scale factor $s_{i}$ and rotation angle $\theta_{i}$ have been determined for $I_{i}$, the global similarity constraint can be expressed as

(1) $$\varphi_{g}\left(V\right)=\sum_{i=1}^{N}\sum_{e_{j}^{i}\in E_{i}}w{\left(e_{j}^{i}\right)}^{2}\left[{\left(c\left(e_{j}^{i}\right)-s_{i}\cos\theta_{i}\right)}^{2}+{\left(s\left(e_{j}^{i}\right)-s_{i}\sin\theta_{i}\right)}^{2}\right].$$

Among them, V represents all grid points, $E_{i}$ represents the edge of image $I_{i}$ grid, $c\left(e_{j}^{i}\right)$ and $s\left(e_{j}^{i}\right)$ are the coefficients of similar transformation, and $w\left(e_{j}^{i}\right)$ is the weight function, which assigns more weight to the mesh farther from the overlapping area. Alignment items play an important role in overlapping areas, for the part far from the overlapping area, because there is no alignment constraint, similarity experience is more important.

3. Local similarity constraint

To reduce overall shape distortion, ensure that overlapping and non-overlapping areas are transformed similarly, the transformation from overlapping regions gradually transits to non-overlapping regions, and the local similarity constraint expression is

(2) $$\varphi_{l}\left(V\right)=\sum_{i=1}^{N}\sum_{\left(j,k\right)\in E_{i}}\|\left({\tilde{v}}_{k}^{i}-{\tilde{v}}_{j}^{i}\right)-S_{jk}^{i}{\left(v_{k}^{i}-v_{j}^{i}\right)\|}^{2}.$$

Among them, $v_{i}$ represents the point of image $I_{i}$ grid, $v_{j}^{i}$ represents the position of original image grid points, and ${\tilde{v}}_{j}^{i}$ represents the position of deformed grid points. $S_{jk}^{i}$ is the similarity transformation of edge $\left(j,k\right)$. We use the similarity transformation equation of [22] to calculate $S_{jk}^{i}$.

4. Line protection constraint

When the scene in the image is not in a plane, the structure information in the image may change significantly by adding a similarity constraint, which affects the subsequent splicing effect. In order to protect the structure of lines in the image, the line protection constraint is introduced into the geometric constraint of overlapping area alignment. We adopt the method in [23] to compute line protection constraint. First, select the local line segments in the image, and then sample them. The line protection constraint can be expressed as

(3) $$\varphi_{l}\left(V\right)=\sum_{l_{k}\in L}\sum_{m_{i}\in M_{k}}\frac{S_{i}}{\left\|v^{\prime }\left(m_{1}\right)-v^{\prime }\left(m_{0}\right)\right\|}.$$

Among them, L is the collection of collected line segments, $m_{0}$ and $m_{1}$ are the two endpoints of the sampled line segments, $v^{\prime }\left(m_{i}\right)$ is the coordinate of the point $m_{i}$, and $S_{i}$ is the quadrilateral area formed by the vector $m_{i}-m_{0}$ and the vector $m_{1}-m_{0}$. $S_{i}$ can be computed as

(4) $$S_{i}=\|\left(v^{\prime }\left(m_{i}\right)-v^{\prime }\left(m_{0}\right)\right)\left(v^{\prime }\left(m_{1}\right)-v^{\prime }\left(m_{0}\right)\right)\|.$$

Based on global, local similarity, and line protection constraints, we construct the cost function to estimate the quality of the deformation mesh. The cost function is

(5) $$f\left(V\right)=\alpha_{1}\varphi_{g}\left(V\right)+\alpha_{2}\varphi_{l}\left(V\right)+\alpha_{3}\varphi_{ll}\left(V\right).$$

Among them, $\alpha_{1}$, $\alpha_{2}$, and $\alpha_{3}$ are weight coefficients of three constraints. We set $\alpha_{1}$, $\alpha_{2}$, and $\alpha_{3}$ to 1, 1, and 1.5, respectively. Our goal is to minimize the cost function to obtain the optimal vertex coordinates of deformable surface mesh. Since all terms in the cost function are quadratic terms, a sparse linear solver is used to solve the optimization problem, finally the optimal vertex set is obtained.

## 4. Experimental Results

In order to verify the effectiveness of proposed aerial image mosaic method, we conducted three groups of experiments. In the first group of experiments, we conducted ablation experiments and compared the image mosaic results of the APAP, AANAP [24] and our method. In the second group of experiments, we compared the stitching results of our method and one of current the most popular UAV image stitching tools, ODM, on color aerial images. In the third group of experiments, we compared the stitching results of the ODM, Pix4D, and our methods on BGNIR multispectral aerial images.

Hardware and software environment configuration in the experiments:

The server: Intel (R) Core (TM) i9-7960X CPU @ 3.30 GHz, Ubuntu 20.04, 64-bit operating system, 32 GB running memory, NVIDIA Corporation GV100 [TITAN V], Python 3.6.

Local computer: Intel (R) Core (TM) i7-10750H CPU @ 2.60 GHz, Windows10, Visual C++11, OpenCV 2.4.0.

### 4.1. Ablation Experiments

**Figure 2:**
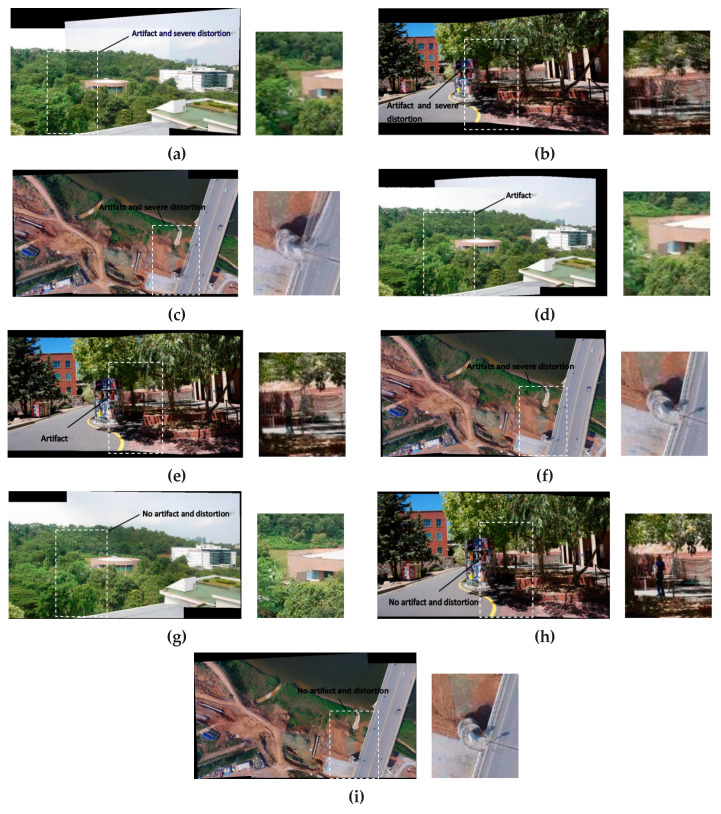
Ablation experiments on Forest, Road, and Bridge dataset. (**a**–**c**) show stitching results of the APAP. (**d**–**f**) show stitching results of the APAP with local and global similarity constraints. (**g**–**i**) show stitching results of the APAP with local, global similarity constraints and line protection constraints (One area of each result is enlarged in the figure).

In order to verify the effectiveness of the global similarity constraint, local similarity constraint and line protection constraint in our method, we have conducted ablation experiments on the existing forest, road, and bridge dataset. The results are provided in Figure 2. The white dotted boxes in stitching result images mark parts of the overlapping region. The small image on the right side of each result image is a part of enlarged region in white dotted boxes. The APAP method is the baseline, and the APAP results are shown in Figure 2a–c with the obvious artifacts and severe distortions in the overlapping areas. The results of the APAP with local and global similarity constraints are shown in Figure 2d–f. The results of Figure 2d,e show distortion issues are improved, but artifacts are non-negligible. The result of Figure 2f still shows artifacts and distortions. The results of the proposed method, incorporate the APAP with local, global similarity constraints and line protection constraints, show artifacts and distortions are effectively suppressed in overlapping area in Figure 2g–i.

We have computed the peak signal to noise ratio (PSNR), structural similarity (SSIM), and root mean square error (RMSE) of the enlarged areas in Figure 2. The best results are obtained when the APAP combines local, global similarity constraints and line protection constraints. The quantitative data are shown in Table 1. The ablation experiment proves that our method is effective.

**Figure 3:**
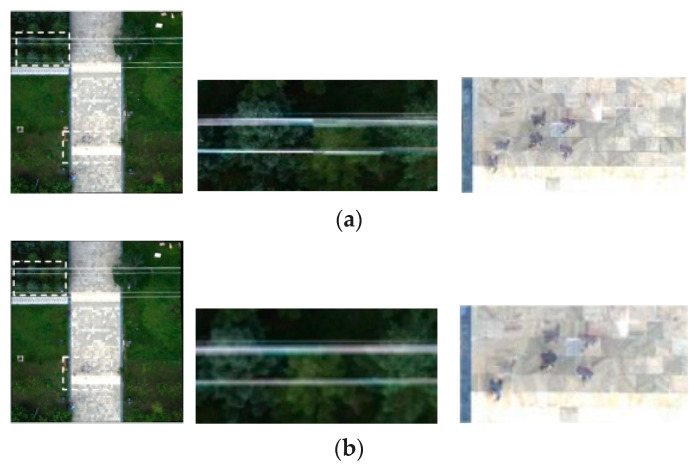

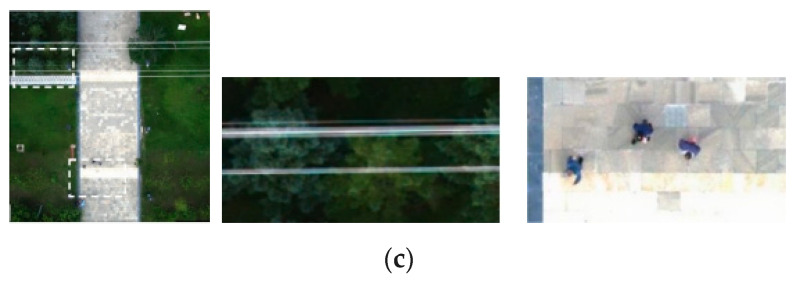
Comparison of Image Mosaic Results. (**a**–**c**) show stitching results of the APAP, AANAP, and ours separately. (**a**) Stitching result of the APAP; (**b**) Stitching result of the AANAP; (**c**) Stitching result of Ours (Two areas of each result are enlarged in the figure).

We also have conducted comparative experiments of proposed algorithm on the images with straight lines to further verify the effect. The compared methods include the APAP and AANAP. The results of the APAP, AANAP, and proposed methods are shown in Figure 3. Two areas of each result have been enlarged. For the results of the APAP and AANAP methods, the artifact and perspective distortion on the straight line and pedestrians on the road are non-negligible. However, our method can successfully mitigate the artifact and perspective distortion.

### 4.2. Construction of UAV Aerial Photography Dataset

**Figure 4:**
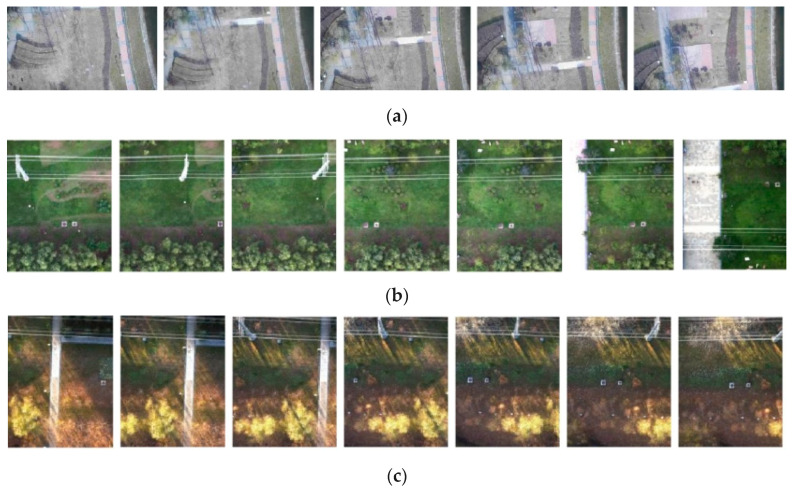
Color aerial dataset images. (**a**) color aerial images in spring (1920 × 1080 pixels); (**b**) color aerial images in summer (1472 × 1852 pixels); (**c**) color aerial images in autumn (1472 × 1852 pixels).

**Figure 5:**
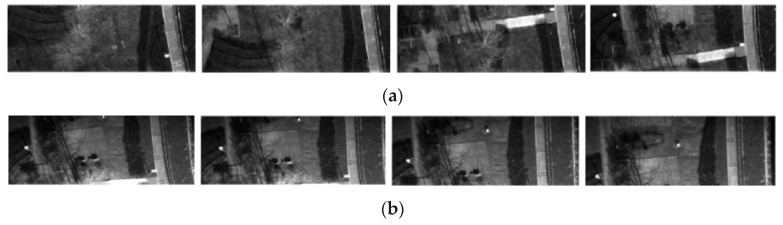

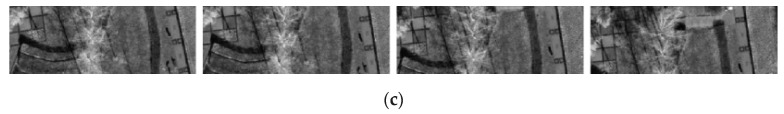
BGNIR multispectral aerial dataset images. (**a**) Blue spectrum images (2160 × 750 pixels); (**b**) Green spectrum images (2160 × 940 pixels); (**c**) Near infrared spectrum images (2160 × 796 pixels).

We also obtained the dataset from UAV aerial photography. The UAV aerial image dataset includes aerial images in spring, summer, autumn, and BGNIR multispectral aerial images. Figure 4a–c show some real color aerial images, and Figure 5a–c show some real BGNIR multispectral aerial images.

### 4.3. Color Aerial Image Stitching

**Figure 6:**
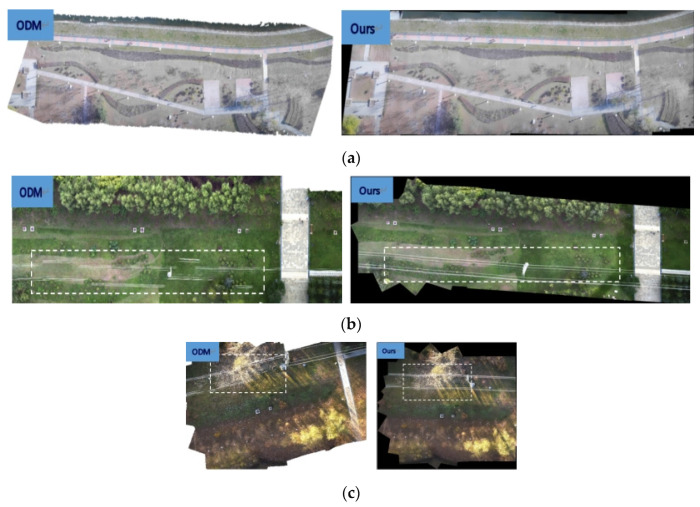
Panoramas of ODM method and our method on real color aerial dataset. (**a**) Panoramas for ODM and ours on spring aerial images; (**b**) Panoramas for ODM and ours on summer aerial images; (**c**) Panoramas for ODM and ours on autumn aerial images.

We used ODM and our method to conduct color aerial image stitching experiment, stitching 20 adjacent frames into a panoramic image. Experiments were conducted on the images of spring, summer, and autumn in the dataset. The experimental results are shown in Figure 6.

The stitching results of ODM and our methods in Figure 6a look well visually. The splicing effect of the two splicing methods on the spring aerial images is similar in Figure 6a. We use white dotted boxes to tag the stitching results of lines in panoramas for ODM and our methods on the summer and autumn aerial images in Figure 6b,c. The results of ODM method in Figure 6b,c show obvious artifacts and distortions of straight-line information due to parallax exist. In contrast, the results of our method in Figure 6b,c can protect the lines in the images and successfully handle the parallax issue. Our method works well and maintains the integrity of straight-line contents.

We quantitatively analyze the two stitching methods using PSNR, SSIM, and RMSE. The specific data are in Table 2, Table 3 and Table 4.

PSNR is an evaluation index based on pixel error, and the higher the peak signal to noise ratio, the better the method used. From the comparison of experimental data in Table 2, the PSNR value of our method is greater than that of ODM method.

The larger the SSIM value, the smaller the difference between the output image and the undistorted image, that is, the better the image quality. From the experimental data in Table 3, our method has less distortion and better stitching effect.

RMSE is an evaluation index to judge the image quality. The smaller the value, the higher the registration accuracy. From the experimental data in Table 4, the quality of our method is better than that of ODM method.

### 4.4. BGNIR Multispectral Aerial Image Stitching

Multispectral image refers to the acquisition of multiple single bands of ground object radiation, and the obtained data will contain spectral information of multiple bands.

**Figure 7:**
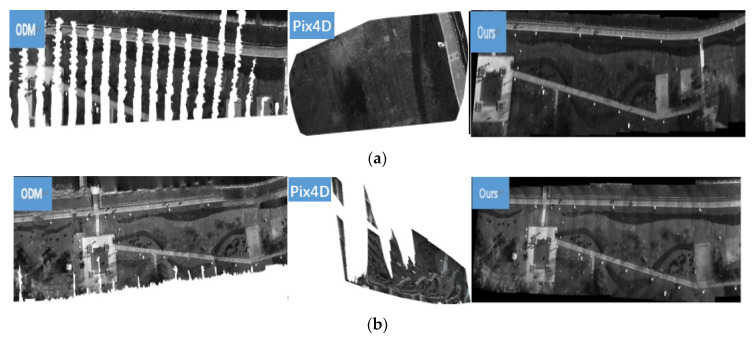

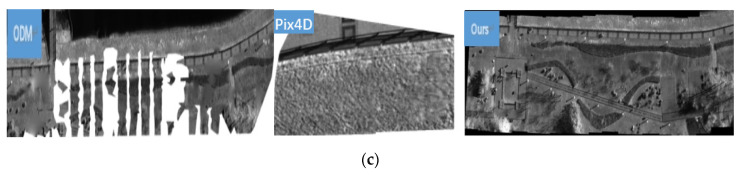
Stitching results of ODM, Pix4D, and proposed method on BGNIR multispectral aerial image dataset. (**a**) Panoramas for ODM, Pix4D, and ours on blue spectrum aerial images; (**b**) Panoramas for ODM, Pix4D, and ours on green spectrum aerial images; (**c**) Panoramas for ODM, Pix4D, and ours on near infrared spectral aerial images.

In this part of the experiment, ODM, Pix4D, and our method are employed to stitch the BGNIR multispectral aerial images, respectively. Through the experiment, the ODM method cannot stitch without pose information, and the ODM with pose information could stitch successfully, but the effect was relatively poor. Similarly, the Pix4D method also cannot stitch without pose information, and the Pix4D with pose information can stitch, but the results are the worst of all methods. Some experimental results are shown in Figure 7.

From Figure 7, we find that our method provides a natural look to the panorama even position and attitude information of UAV are not used. There are no visible parallax errors and perspective distortions. In contrast, obvious distortions, artifacts, and information loss occur in the results of ODM and Pix4D with position and attitude information.

Similarly, we use PSNR, SSIM, and RMSE to quantitatively analyze the results of the three methods in BGNIR multispectral dataset. Due to the stitching results of Pix4D incomplete, we cannot calculate PSNR, SSIM, and RMSE values of them. The specific data are shown in Table 5, Table 6 and Table 7.

From the experimental data in Table 5, Table 6 and Table 7, our method is superior to ODM in PSNR, SSIM, and RMSE values.

### 4.5. Running Time

The stitching running time of ODM, Pix4D, and our method is calculated, respectively. The results are shown in Table 8. The speed of our method is relatively fast, whether in color aerial images or BGNIR multispectral aerial images. In the process of color aerial image stitching, ODM and our methods can perform stitching without position and attitude information, and the ODM splicing speed is a little slower. When ODM and Pix4D methods do not use position and attitude information on BGNIR multispectral aerial image, the stitching fails, and the splicing time with position information is about twice that of our method. Therefore, our method is the fastest of threes methods.

## 5. Discussion

In order to solve the problems of artifacts and distortions in the mosaic of aerial images with certain parallax, and mosaic failure of multispectral aerial images due to fewer feature points, we use AKAZE algorithm to extract feature points of aerial images, and add line protection constraints, global, and local similarity constraints to protect image structure information based on APAP, and finally obtain panoramic aerial image. Our method has good results when stitching real color aerial images and BGNIR multispectral aerial images. The experimental results demonstrate that our method performs well in artifact and distortion suppression and stitching time. The proposed stitching method has practical application value in the field of land resource survey and environmental monitoring.

## Figures and Tables

**Table 1:** Numerical Results of Ablation Experiment.

Images	Constraints			Evaluation Data		
	Local Similarity	Global Similarity	Straight Line Protection	PSNR	SSIM	RMSE
Forest image	\	\	\	13.542	0.119	2.876
	√	√	\	15.454	0.130	1.768
	√	√	√	15.491	0.146	1.714
Road image	\	\	\	10.100	0.044	6.353
	√	√	\	10.346	0.043	6.004
	√	√	√	11.601	0.060	4.497
Bridge image	\	\	\	15.781	0.237	1.718
	√	√	\	15.368	0.255	1.889
	√	√	√	15.886	0.267	1.677

**Table 2:** PSNR values of two methods on color aerial image mosaic.

Methods	Spring	Summer	Autumn
ODM	14.6241	15.0827	14.3579
Ours	14.6612	16.8629	16.2582

**Table 3:** SSIM values of two methods on color aerial image mosaic.

Methods	Spring	Summer	Autumn
ODM	0.1722	0.2057	0.2019
Ours	0.2246	0.3297	0.2051

**Table 4:** RMSE values of two methods on color aerial image mosaic.

Methods	Spring	Summer	Autumn
ODM	2.242	2.379	2.384
Ours	2.223	1.339	1.539

**Table 5:** PSNR values of ODM and our methods on BGNIR multispectral aerial image mosaic.

Method	Blue Spectrum	Green Spectrum	Near Infrared Spectrum
ODM	13.6184	14.3390	14.3967
Ours	18.1874	18.4552	14.7783

**Table 6:** SSIM values of ODM and our methods on BGNIR multispectral aerial image mosaic.

Method	Blue Spectrum	Green Spectrum	Near Infrared Spectrum
ODM	0.4596	0.4285	0.2148
Ours	0.5806	0.5914	0.3210

**Table 7:** RMSE values of ODM and our methods on BGNIR multispectral aerial image mosaic.

Method	Blue Spectrum	Green Spectrum	Near Infrared Spectrum
ODM	2.826	2.394	2.363
Ours	1.066	1.029	2.164

**Table 8:** Stitching time of two methods on aerial images mosaic (s).

Methods	Spring	Summer	Autumn	Blue Spectrum	Green Spectrum	Near Infrared Spectrum
ODM	34.33	44.87	38.64	63.45	72.43	70.36
Pix4D	\	\	\	64.02	113.97	61.01
Ours	21.99	35.15	25.95	30.23	37.16	37.18

## Data Availability

The datasets presented in this study are available from the corresponding authors upon reasonable request.
